# Evaluation and Verification of the Global Rapid Identification of Threats System for Infectious Diseases in Textual Data Sources

**DOI:** 10.1155/2016/5080746

**Published:** 2016-09-06

**Authors:** Andrew G. Huff, Nathan Breit, Toph Allen, Karissa Whiting, Christopher Kiley

**Affiliations:** ^1^Michigan State University, 736 Wilson Road, East Lansing, MI 48824, USA; ^2^EcoHealth Alliance, New York, NY, USA; ^3^Defense Threat Reduction Agency, Fort Belvoir, VA, USA

## Abstract

The Global Rapid Identification of Threats System (GRITS) is a biosurveillance application that enables infectious disease analysts to monitor nontraditional information sources (e.g., social media, online news outlets, ProMED-mail reports, and blogs) for infectious disease threats. GRITS analyzes these textual data sources by identifying, extracting, and succinctly visualizing epidemiologic information and suggests potentially associated infectious diseases. This manuscript evaluates and verifies the diagnoses that GRITS performs and discusses novel aspects of the software package. Via GRITS' web interface, infectious disease analysts can examine dynamic visualizations of GRITS' analyses and explore historical infectious disease emergence events. The GRITS API can be used to continuously analyze information feeds, and the API enables GRITS technology to be easily incorporated into other biosurveillance systems. GRITS is a flexible tool that can be modified to conduct sophisticated medical report triaging, expanded to include customized alert systems, and tailored to address other biosurveillance needs.

## 1. Introduction

Infectious diseases pose a significant threat to global health and economic stability [1, 2]. Due to extensive globalization and urbanization, infectious diseases can spread at unprecedented rates [3]. Small and localized infectious disease threats can rapidly become international catastrophes, as demonstrated by influenza (H1N1A) in 2009, Ebola Virus Disease in 2014, and Middle Eastern Respiratory Syndrome in South Korea and the Middle East [4–6]. Identifying infectious disease outbreaks is critical to reducing overall harm and preventing epidemics. Increasing biosurveillance systems' detection and communication speed may contribute to a reduction in overall health and economic consequences from infectious diseases.

Traditional biosurveillance systems rely predominantly on local clinicians, laboratory technicians, and public health practitioners to identify infectious disease outbreaks. In part, these systems identify cases via routine patient care where samples are collected. Then, laboratory testing is performed on the collected samples, and clinical case definitions are established. Via these processes, infectious disease cases are typically reported to a centralized authority that then aggregates and monitors cases for signs of an above normal caseload.

Unfortunately, traditional biosurveillance systems are limited by their cost, their limited geographic coverage, and their inability to rapidly communicate results. For example, an upgrade to the existing United States' Biowatch program costs 61 million dollars and was canceled before its completion. Furthermore, effective biosurveillance systems depend on the quality of underlying health care infrastructure, which can be highly variable geographically. Without a vast network of healthcare infrastructure, traditional biosurveillance systems may not be sensitive enough to detect rare infectious diseases. Also, some traditional biosurveillance systems may not be accurate (e.g., lack of laboratory capacity), thereby overwhelming the infectious disease analyst with incorrect or irrelevant information.

In part, these barriers lead to incomplete geographic coverage, varying by disease type, for traditional surveillance systems. As a direct result of incomplete geographic coverage for some infectious diseases, infectious disease outbreaks in regions with inadequate healthcare infrastructure may not be identified in the outbreak's early stages, as seen with the ongoing Ebola Virus Disease epidemic in West Africa (not identified as EVD until 85 days after the first case). Governments, who may be reticent to announce an outbreak for fear of economic harm, often control traditional biosurveillance systems as occurred with the 2003 SARS outbreak. With current healthcare technology and investment levels traditional biosurveillance systems lack complete coverage.

Typically, traditional biosurveillance systems are tailored to a single infectious disease (e.g., ILInet, Malaria Early Warning System, and European Legionnaire's Disease Surveillance Network), requiring clinicians to report diseases based on predefined lists [7]. Different governing entities have different lists of infectious diseases that must be reported by clinicians, and these lists are at times updated to reflect the current needs of the public health community. In some cases, traditional biosurveillance capabilities are implemented for specific classes of diseases, transmission pathways, and specialized laboratory capabilities (e.g., ILInet, Foodnet, and Pulsenet). Most traditional biosurveillance systems are well suited to monitor known infectious disease threats (e.g., poliovirus, influenza) but are not designed to detect threats from unknown or extremely rare infectious diseases [8].

The term syndromic surveillance is used to refer to a number of different types of biosurveillance systems where symptoms are used to classify the type of infectious diseases [8]. Syndromic surveillance was first used to describe biosurveillance cases that conformed to particular clinical case definitions (this is especially useful when monitoring diseases where no laboratory test exists). However, its usage has expanded to encompass most forms of biosurveillance outside of traditional biosurveillance systems. These include systems that collect information on hospital admissions, pharmaceutical sales, employee absenteeism, and other data streams that are used to detect outbreaks [8].

Digital disease detection, also called digital biosurveillance, refers to analysis of web data for insight on public health and infectious disease systems [8]. The term is broadly defined to include various uses of web-native information: (1) aggregation of medical reports from subject matter experts (e.g., ProMED-mail); (2) computational models built upon search results and web traffic (e.g., Google Flu Trends); and (3) models built on other clusters of search terms around infectious disease trends. Digital biosurveillance examines indirect evidence for infectious disease cases (e.g., textual data sources from symptomatic people) and must work in combination with traditional biosurveillance methods. Digital biosurveillance's greatest potential is that it can possibly identify potential outbreaks where traditional biosurveillance systems do not exist and can rapidly detect and communicate infectious disease outbreaks.

Digital biosurveillance holds promise but has yet to fulfill a concrete role as an early warning system in public health biosurveillance. There is disagreement about the utility of digital disease surveillance for predicting influenza outbreaks [9, 10]. Initially, there was some evidence that Google Flu Trends was useful in forecasting developing influenza outbreaks [8]; however, Google Flu Trends was inconsistently accurate from year to year and there were substantial flaws in Google Flu Trends ability to predict regular seasonal influenza peaks and irregular pandemic influenza [10]. Furthermore, tools and analytical methods that rely upon human curation of data feeds (e.g., ProMED-mail, HealthMap) require significant human capital and appear to scale with the amount of training and education of the human curators [11].

Despite these weaknesses of digital disease surveillance, natural language processing (NLP) is a potentially useful tool for biosurveillance systems. NLP is able to give structure to unstructured textual data. For example, NLP has been used to automatically classify electronic medical records (EMR) from emergency rooms into categories for syndromic biosurveillance [12, 13], especially in cases where specific clinical definitions are scant (e.g., invasive mold) [14]. In the realm of digital biosurveillance, efforts are underway to apply NLP to social media streams [15]. Using NLP to systematically create structured data from unstructured text may enable the monitoring of innumerable local sources of infectious disease information globally. Digital biosurveillance methods that use NLP may lead to the accurate and rapid detection of infectious disease outbreaks in places where traditional biosurveillance systems are insufficient. For these reasons, EcoHealth Alliance developed the Global Rapid Identification of Threats System (GRITS) that uses NLP to identify emerging infectious disease threats in textual sources.

## 2. Method

GRITS uses natural language processing to determine which infectious diseases are most likely associated with an input text sample. Articles are processed using a combination of NLP methods to identify disease-related features from the text. These features are passed to an ensemble of binary logistic regression classifiers, which work together to “diagnose” the article, ranking diseases by predicted probability.

### 2.1. GRITS' Search Function

GRITS presently searches an index of over 250,000 infectious diseases related articles. Elasticsearch assigns relevance scores to individual terms using TF-IDF (term frequency-inverse document frequency) based models, which weight matches according to how common words are in a document divided by how rare they are across the corpus of documents. Additionally, GRITS' extracted feature metadata for each article (including infectious disease keywords, date, and location) are searchable can be used to sort search results.

### 2.2. Feature Extraction

GRITS' text processing and NLP algorithms, written mainly in Python, extract disease-related and contextual features from texts and store these features as annotations on the text. The algorithms are detailed in Supporting Information, and code samples are provided (Supplemental 1 in Supplementary Material available online at http://dx.doi.org/10.1155/2016/5080746). Prior to analysis, non-English text is translated using Bing Translator.

Feature extraction is performed using Python's standard pattern-matching libraries and the NLTK package to match keywords from a variety of compiled ontologies of terms related to infectious disease and public health (Table 1).

Features are categorized: diseases, pathogens, symptoms, hosts, and modes of transmission. Dates are extracted with the Stanford SUTime Java library. Locations are matched with a custom algorithm that uses data from the GeoNames database in addition to a number of heuristics to reduce false positive matches. Case counts are identified using the CLiPS Pattern library's search module, with a number of specifically tailored search phrases. GRITS stores extracted features in JSON with information about their position in the text, so they can be viewed separately from the document or in their original context.

### 2.3. Classifier Training, Verification, and Evaluation

GRITS uses the binary relevance method (as implemented in scikit-learn's sklearn.multiclass.OneVsRestClassifier) to predict the disease referred to by a body of text. This uses an ensemble of logistic regression classifiers, one for each disease label (approximately 120). Each classifier estimates the probability that a text passage is associated with a single disease, given the vector of features extracted by GRITS' NLP algorithms. Multiple occurrences of features were not counted.

Classifier training and testing used a randomly selected corpus of approximately 150,000 articles from a 2 to 3-year period (of the 250,000 article set) and collected and assigned a single disease label each by analysts. Classifiers were trained on a subset of approximately 12,000 articles. Each classifier fits a logistic regression model, using articles with that classifier's disease label as positive responses and all other articles in the training set as negative responses.

## 3. Results

### 3.1. GRITS' Diagnostic Performance Evaluation

The classifiers' performance was tested over a set of approximately 3500 health news articles and ProMED reports. A confusion matrix was composed, from which the microaveraged F1 score was calculated across all classifiers. The microaveraged F1 score sums all true positives, false negatives, and false positives, evaluating classifier performance across all diseases in the GRITS ontology. To determine the relative contribution of features for a given diagnosis on a text, the regression coefficients for each classifier are rescaled to sum to 1 and then multiplied by the estimated probability of that disease for that text.

### 3.2. GRITS' Diagnostic Algorithm Verification

The GRITS disease classification system has an overall precision (positive predictive value) of 64% and recall (sensitivity) of 63%. The overall F1 score is 0.317. However, GRITS diagnoses some diseases very well (Table 2) and some diseases very poorly (Table 3). These results included translations and were not skewed due to translation.

## 4. Discussion

### 4.1. Context for Biosurveillance

GRITS provides a framework for classifying the infectious disease-related content in potentially arbitrary text. Monitoring digital disease signals for impending infectious disease threats means that biosurveillance capacity can be extended to areas where the healthcare and public health infrastructure is inadequate. This is crucial since many emerging infectious disease threats occur in places where traditional biosurveillance infrastructure is scant.

In the hands of the astute public health analyst, GRITS is a powerful tool for infectious disease biosurveillance that allows users to efficiently monitor nontraditional data sources for infectious disease threats. It can extend the capabilities of analysts to triage and monitor a wider range of textual sources, increasing coverage of nontraditional digital disease surveillance in areas where traditional systems do not exist and supplementing traditional methods where they do. GRITS is currently used in the Defense Threat Reduction Agency's (DTRA's) Biosurveillance Ecosystem (BSVE) to identify infectious disease threats globally.

### 4.2. Limitations and Future Directions

Large sources of annotated disease-related textual data, required to accurately train machine learning classifiers, are uncommon, difficult to come by, and time-consuming to create. The HealthMap data used to train the GRITS classifiers is sufficiently large, but each article is only labeled with one disease, even when a text may mention multiple diseases. This means that disease traits extracted from an article may not map specifically to the disease that article is labeled with, negatively impacting classifier training.

The HealthMap training data consists largely of aggregated online news articles, WHO, and ProMED-mail reports. These texts have a set of features linguistic properties specific to online news articles related to health. If GRITS were applied to other sources of text, like scholarly articles or social media feeds, new sets of training data would likely have to be curated.

In an active surveillance system using GRITS, feature ontologies and article classifiers should be updated on an ongoing basis. New diseases will emerge, disease classifications and ontologies will change, and the GRITS system must be updated to prevent diminishing accuracy. Incorporating feedback from GRITS users (from the results of individual articles) into classifier training would improve classifier fit for that article type.

GRITS currently exists as a standalone web application. However, its utility would be increased as part of a larger suite of biosurveillance tools and with connections to continuous data feeds. These would enable building out various decision support capabilities around the GRITS toolset. For instance, GRITS could store processed text sources and display summaries of articles temporally, spatially, or by diagnosed disease or public health keyword. An alert system could be built on top of this dataset to warn users of potentially dangerous clusters of reports, and additional ontologies could be created to train GRITS to make educated conclusions on additional complex variables like pathogen class, report risk level, or the emergence of a novel pathogen. Additionally, through the GRITS API, these tools are planned for incorporation to the Defense Threat Reduction Agency's Biosurveillance Ecosystem (BSVE) and will run continuously on BSVE data feeds [16].

## Supplementary Material

Supplemental 1 contains links to all of the source code and key data repositories used in this study.

## Figures and Tables

**Table 1:** The ontologies used in GRITS, their contents, and their descriptions.

Ontology	Contents	Description
Biocaster ontology	General disease ontology	English terms for symptoms, diseases, and pathogens are used as features

GRITS ontology	Curated ontology of symptoms, control measures, descriptions of infected individuals, diseases, disease categories, environmental factors, hosts, host uses, modes of disease transmission, occupations, disease risks, vectors, and zoonotic types	Collection of keywords and terms gathered and vetted from a consensus of experts at EcoHealth Alliance

HealthMap disease labels	Diseases identified as significant by HealthMap and used for their disease labels	Used as outcome in logistic regression models

The disease ontology	Human disease related terms, phenotypic characteristics, and medical vocabulary disease concepts	Disease names and synonyms are used as keyword features. Predicates from disease definitions

USGS topographic feature vocabularies	Environmental factors	Subset used as features (all labels and synonyms of type owl#Thing)

Wordnet	English language ontology that maps word relatedness	Hyponyms and lemmata for a set of epidemiology-related root keywords are used as features

**Table 2:** GRITS' top 10 performing disease classifications.

Disease	Precision (PPV)	Recall (sensitivity)	*F*1 score	*N* of positive articles
Avian influenza	0.923	0.932	0.928	208
Hepatitis	0.989	0.866	0.923	112
Influenza	0.905	0.959	0.931	830
Listeriosis	0.921	0.951	0.936	62
Measles	0.931	0.964	0.947	226
Polio	0.893	0.976	0.933	43
Salmonella	0.871	0.983	0.924	124
Scabies	1	0.862	0.925	29
Syphilis	0.928	0.928	0.928	14
Tuberculosis	0.939	0.951	0.945	82

**Table 3:** GRITS' bottom 10 performing disease classifications.

Disease	Precision (PPV)	Recall (sensitivity)	*F*1 score	*N* of positive articles
Rubella	1	0.09	0.166	11
Respiratory illness	0.666	0.181	0.285	11
Campylobacter	0.875	0.538	0.666	13
Chikungunya	0.651	0.823	0.727	34
*Clostridium difficile*	0.888	0.235	0.372	34
Eastern equine encephalitis	0.833	0.416	0.555	12
Hemorrhagic fever	0.486	0.947	0.642	19
HIV/AIDS	0.687	0.733	0.709	15
Lyme disease	0.588	0.833	0.689	12
*Neisseria meningitidis*	0.707	0.659	0.682	44
