# Supplementary material for: Evaluation and Verification of the Global Rapid Identification of Threats System for Infectious Diseases in Textual Data Sources
Source: Interdiscip Perspect Infect Dis. 2016 Sep 6;2016:5080746. doi: 10.1155/2016/5080746 (PMC5028852; doi:10.1155/2016/5080746)
Supplement: Supplementary file 1 — Supplemental 1 contains links to all of the source code and key data repositories used in this study. [file 5080746.f1.docx]

Supplemental 1

# Access to GitHub Files

GRITS’s code is available on GitHub across a number of repositories under the Apache 2.0 license. Code may be downloaded as ZIP files or cloned using Git (at time of writing, instructions are available at <https://help.github.com/articles/set-up-git/>).

# Key GRITS Repositories

Key repositories containing GRITS code include:

- Annie, a set of Python modules for document annotation: <https://github.com/ecohealthalliance/annie>
- grits-api, the back-end for the GRITS website: <https://github.com/ecohealthalliance/grits-api>
- GRITS Diagnostic Dashboard, the front-end for the GRITS website: <https://github.com/ecohealthalliance/diagnostic-dashboard>
- grits-deploy-ansible, a repo which provides installation scripts for the GRITS suite: <https://github.com/ecohealthalliance/grits-deploy-ansible>
